# Surface marker-defined functional subpopulations of mesenchymal stromal cells: mechanisms, applications, and clinical translation

**DOI:** 10.3389/fbioe.2026.1911125

**Published:** 2026-09-24

**Authors:** Shuang Wu, Shengmeng Yuan, Jiting Li, Wenqi Sun, Fangjun Huo, Lei Liu, Chao Yang, Weidong Tian

**Affiliations:** 1 Department of Oral and Maxillofacial Surgery, West China Hospital of Stomatology, Sichuan University Chengdu, Chengdu, Sichuan, China; 2 State Key Laboratory of Oral Diseases and National Clinical Research Center for Oral Diseases and Engineering Research Center of Oral Translational Medicine, Ministry of Education and National Engineering Laboratory for Oral Regenerative Medicine, West China Hospital of Stomatology, Sichuan University, Chengdu, Sichuan, China; 3 Chengdu Shiliankangjian Biotechnology Co., Ltd., Chengdu, Sichuan, China; 4 Sichuan Tianfu Cell Quality Detection and Evaluation Center Co., Ltd., Chengdu, Sichuan, China

**Keywords:** cell sorting, functional markers, mesenchymal stem cells, subpopulation heterogeneity, tissue regeneration

## Abstract

Mesenchymal stromal cells (MSCs) hold considerable promise in tissue regeneration due to their multilineage differentiation, paracrine signaling, and immunomodulatory capabilities, as evidenced by thousands of clinical trials. However, clinical translation is constrained by inconsistent and unpredictable efficacy. This primarily stems from MSC heterogeneity, including differences in tissue origin, functional subpopulations, donor age and disease status, and phenotypic drift and functional decline during *in vitro* expansion. Traditional markers recommended by the International Society for Cell Therapy (ISCT), such as CD73, CD90, and CD105, define MSC identity but cannot discriminate functional subpopulations for angiogenesis, bone repair, or immunomodulation. Thus, the absence of functional surface markers has become a fundamental obstacle to precision MSC therapy and robust quality control. Identifying and harnessing functional surface markers that enrich specific regenerative subpopulations represents a key strategy for overcoming heterogeneity and improving the predictability of clinical outcomes. This review focuses on bone and cartilage, adipose, vascular, neural, muscle, and epithelial regeneration. It systematically examines the mechanisms of action and therapeutic applications of these markers and their subpopulations, and discusses the challenges and future directions for clinical translation.

## Graphical Abstract

**Figure:**
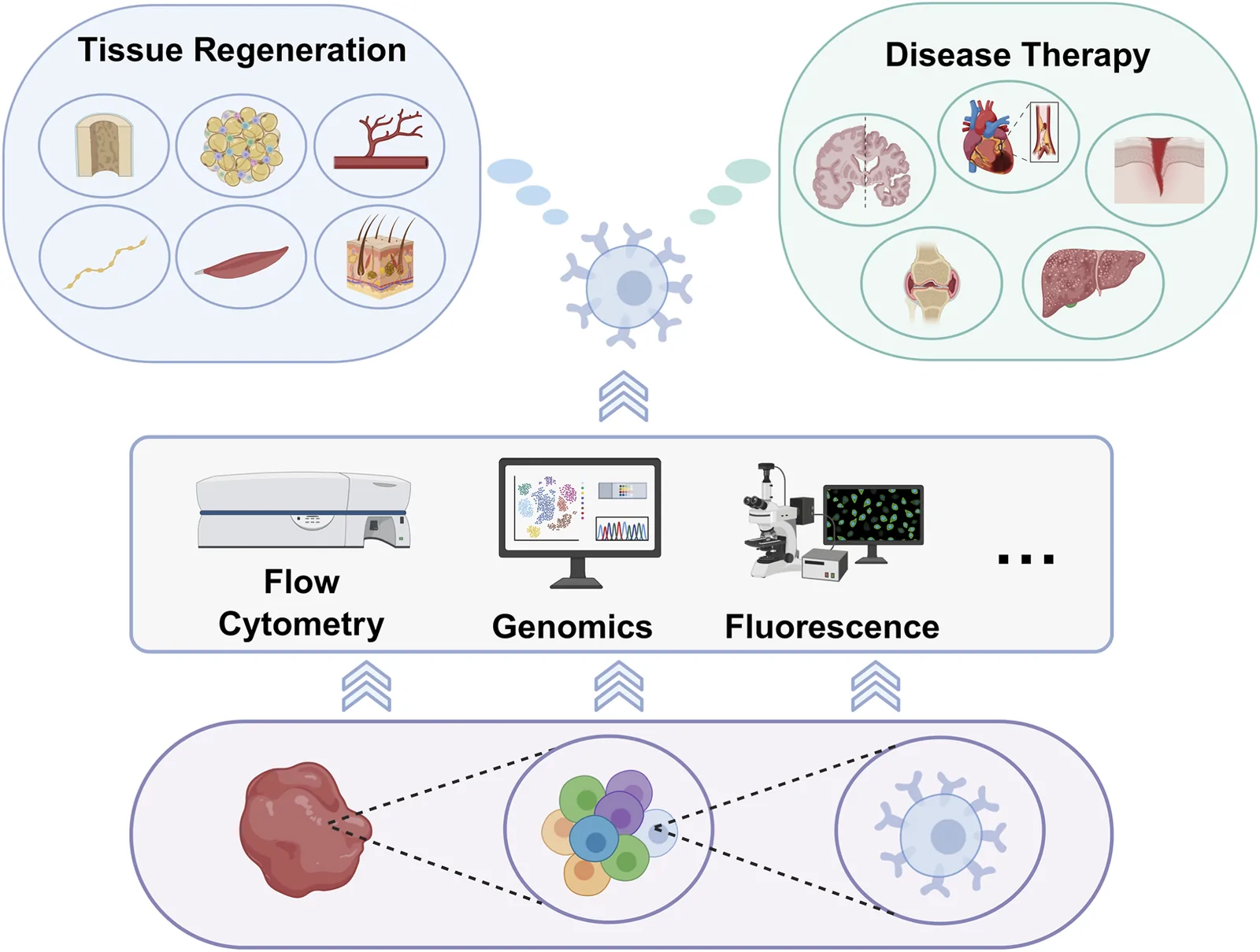
This graphical abstract depicts the screening of functional MSC subpopulations (bottom), the use of advanced technologies (middle), and the research and application of marker-sorted subsets in six regeneration types (left) and five diseases (right), highlighting the discovery-to-therapy continuum.

## 1 Introduction

Major diseases including osteoarthritis, spinal cord injury, myocardial infarction, and diabetic foot ulcers are marked by high disability and mortality due to limited intrinsic tissue repair capacity. Artificial joint replacement, revascularization, and conventional wound care rarely achieve functional tissue regeneration (Bardill et al., 2022; Lawton et al., 2022; Yeo et al., 2022). Liver transplantation, a curative approach for end-stage organ failure, is severely limited by donor scarcity (Kwong et al., 2024). There is an urgent need for novel regenerative strategies that can restore tissue function.

Mesenchymal stromal cells (MSCs) are core seed cells in regenerative medicine due to their multilineage differentiation, paracrine, and immunomodulatory properties. They secrete bioactive molecules such as vascular endothelial growth factor (VEGF) and hepatocyte growth factor (HGF) to promote angiogenesis and suppress inflammation and fibrosis, and can differentiate into osteogenic, chondrogenic, and neural lineages. Over 2,000 clinical trials have been registered and more than 17 products approved for osteoarthritis, graft-versus-host disease, Crohn’s disease, and spinal cord injury, demonstrating significant clinical potential (Galipeau and Sensébé, 2018; Hoang et al., 2022). MSCs therapy has thus advanced rapidly to clinical application, becoming a key pillar in overcoming regenerative bottlenecks.

Nevertheless, clinical translation of MSCs therapy is hampered by inconsistent and poorly predictable efficacy. Although safety is well established, efficacy endpoints vary considerably owing to MSCs heterogeneity (Wang et al., 2021a). This heterogeneity encompasses differences in tissue origin, functionally biased subpopulations within the same tissue, donor age and disease status, and *in vitro* expansion conditions, all causing marked shifts in cellular function (Li et al., 2023). Current ISCT markers (CD73, CD90, CD105) confirm mesenchymal identity but cannot distinguish functional subpopulations responsible for angiogenesis, bone repair, or immunomodulation.

Functional surface markers that specifically identify and isolate MSC subpopulations with defined regenerative properties offer a key strategy to overcome heterogeneity. Given the direct link between these markers and subpopulation behavior, flow cytometry or magnetic bead enrichment enables the recovery of high-purity functional subsets, thereby improving product uniformity (Murray et al., 2017). For example, LIFR^+^/PDGFRβ^+^ cells exhibit enhanced osteogenesis for bone repair (Zhang, P. et al., 2023); CD146^+^ cells are potently pro-angiogenic and promising for ischemia (Bermudez et al., 2025); CD271^+^ cells possess multilineage potential (Poloni et al., 2009). These examples provide preliminary validation of the feasibility of functional marker-driven precision therapy.

Single-cell RNA sequencing has propelled the systematic discovery of functional markers. Researchers have identified stemness, lineage-biased, and proliferative subpopulations in bone marrow MSCs and constructed cross-tissue atlases spanning over 130,000 cells (Xie et al., 2022). Notably, LepR^+^ and Grem1^+^ subsets are core skeletal stromal cells essential for bone homeostasis (Zhou et al., 2014; Worthley et al., 2015); CD200^+^ subsets show strong immunomodulation relevant to inflammatory diseases (Ding et al., 2025). Databases like MSCs DB facilitate heterogeneity analyses. Despite discoveries in bone, vascular, skin, and neural regeneration, a comprehensive review integrating mechanisms, subset features, and therapeutic evidence by regeneration type and disease is lacking.

This review examines six major types of regeneration: osteogenesis and chondrogenesis, adipogenesis, angiogenesis, neurogenesis, myogenesis and epitheliogenesis. It systematically evaluates the mechanisms of functional surface markers and their subpopulations, and summarizes therapeutic progress in neurological diseases, circulatory system disorders, skin and mucosal wounds, bone and joint diseases and hepatobiliary diseases. Finally, it discusses challenges including cross-tissue marker consistency, sorting technology standardization, and clinical translation, aiming to inform the development of precision MSCs therapy.

## 2 MSCs and their multidimensional heterogeneity

MSCs are adult multipotent stromal cells, isolated from tissues such as bone marrow, adipose tissue, and umbilical cord, that possess self-renewal and multilineage differentiation capacities (Guo et al., 2020b; Timaner et al., 2020; Wang et al., 2021b; Yu et al., 2025). Their therapeutic utility stems from three properties: differentiation into other cells such as osteoblasts, chondrocytes, and adipocytes for structural repair; paracrine secretion of VEGF, HGF, and PGE2 that promotes angiogenesis, suppresses inflammation and apoptosis, and creates a pro-repair microenvironment; and low immunogenicity coupled with immunomodulatory capacity to evade allogeneic rejection and restrain excessive immune responses (Trigo et al., 2025). However, consistent therapeutic performance is severely limited by MSC heterogeneity, making an understanding of this heterogeneity essential to appreciate the value of functional markers.

MSC heterogeneity is first evident in tissue source diversity. MSCs from different anatomical sites differ markedly in surface marker expression, proliferation, and differentiation potential. Bone marrow MSCs highly express CD146 and CD271 and are strongly osteogenic; umbilical cord MSCs highly express OCT4 and SOX2 with superior expansion; adipose MSCs highly express CD36, CD10, and CD200 and exhibit robust adipogenesis; dental pulp MSCs co-express STRO-1 and CD146 with neural potential (Mattei et al., 2025). This tissue-source heterogeneity is further exemplified by Xu et al., who demonstrated that CD146^+^ pericytes from periosteum, adipose, and dermis display profoundly different osteogenic potentials, with periosteal cells exhibiting superior bone formation linked to enriched CXCR4 signaling (Wang et al., 2024c).

Even within a single tissue, MSCs display pronounced subpopulation heterogeneity. Classical markers define populations that remain highly heterogeneous and cannot resolve functionally distinct subsets. For the bone marrow marker LepR, Zhou et al. demonstrated that LepR + cells constitute only 0.3% of nucleated cells yet account for 94% of CFU-F and are the main source of adult bone and marrow fat. Single-cell sequencing later revealed multiple LepR + subpopulations with divergent osteogenic and adipogenic capacities, highlighting the inadequacy of a single marker for functional enrichment (Tikhonova et al., 2019). This phenomenon is not restricted to bone marrow. In adipose tissue, Wang et al. used PDGFRα reporter mice to map perivascular subsets and found that PDGFRα^+^/CD34^+^ adventitial cells localized to the tunica adventitia possess the highest mineralization potential. Other perivascular subpopulations within the same tissue do not exhibit this capacity, illustrating that even within a single tissue, spatially distinct microanatomic compartments harbor functionally divergent MSC subpopulations (Wang et al., 2020).

Single-cell sequencing has substantially advanced understanding of MSC functional heterogeneity, revealing subpopulations biased toward connective tissue regeneration, epithelial repair, myogenesis, and neural support. Zhang et al. identified two functional subsets in fresh umbilical cord MSCs enriched for immunomodulatory and osteo/chondrogenic genes, respectively (Zhang et al., 2021). Another study found five bone marrow MSC subpopulations and validated the immunomodulatory role of CD106^+^ cells (Zhang et al., 2022). Even cells fully compliant with ISCT criteria can be resolved into functionally distinct subsets, confirming at single-cell resolution the insufficiency of traditional markers.

Critically, some ISCT-noncompliant subpopulations exhibit superior therapeutic potential. Cardiac-derived CD90^−^ stromal cells, despite lacking the required CD90 expression, significantly outperform CD90^+^ cells and unsorted MSCs in angiogenesis, anti-inflammation, anti-fibrosis, and myocardial repair (Gambini et al., 2025). This directly demonstrates that ISCT markers not only fail to distinguish functional subsets but may also exclude therapeutically valuable populations, reinforcing the urgency of identifying novel functional markers.

## 3 Functional surface markers of MSCs involved in regeneration of different tissues

MSCs exhibit profound functional heterogeneity, and surface markers have become indispensable for identifying subpopulations with defined regenerative properties. This section provides a comprehensive overview of functional surface markers organized by six key tissue regeneration processes: osteogenesis and chondrogenesis, adipogenesis, angiogenesis, neurogenesis, myogenesis, and epitheliogenesis (Figure 1). For each process, the contributions of classical markers are presented alongside emerging markers discovered through recent advances such as single-cell transcriptomics. By highlighting the mechanisms through which these markers distinguish functional subsets, this section aims to illustrate the translational promise of marker-guided MSC therapies.

**FIGURE 1:**
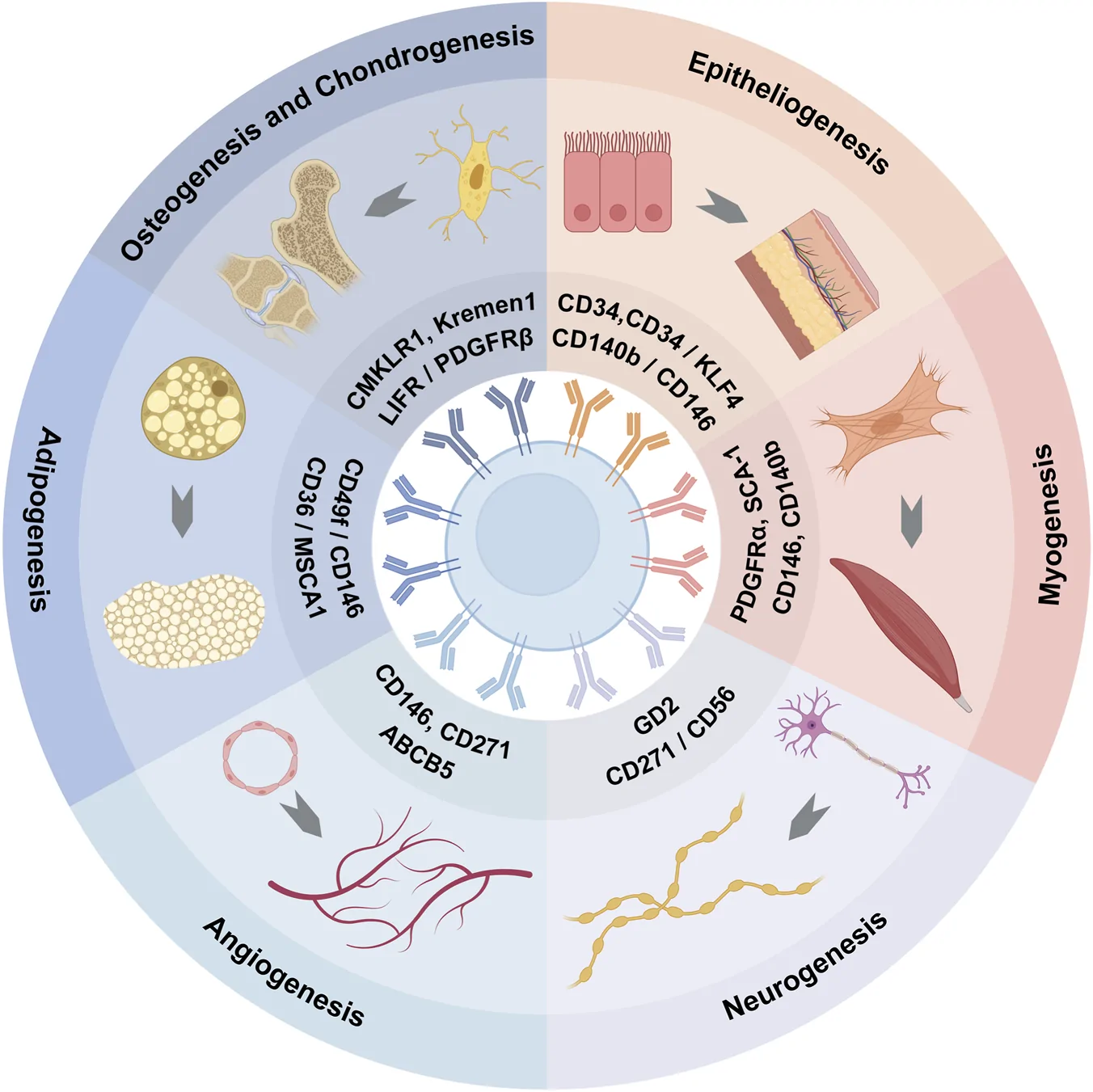
Schematic diagram of functional surface markers of MSCs involved in regeneration of different tissues.

### 3.1 Osteogenesis and chondrogenesis

Surface markers play a pivotal role in identifying MSC subpopulations with osteogenic and chondrogenic potential, as the progression from mesenchymal progenitors to committed osteoblasts or chondrocytes is accompanied by dynamic changes in the expression of distinct membrane proteins. CD56, CD271, CD146, and STRO-1 are classical markers widely used to enrich osteochondral progenitors. CD56^+^ mesenchymal stromal cells represent an osteogenically committed subset, exhibiting significantly higher expression of osteoblast-related genes (Ilas et al., 2020). CD271^+^ subsets display high clonogenicity and robust trilineage differentiation, with demonstrated therapeutic effects in cartilage and bone repair. CD146^+^ cells similarly possess multilineage potential and can be used to select populations with superior bone and cartilage repair capacity (Xie, A. et al., 2021; Miteva et al., 2025). STRO-1, one of the earliest markers for bone marrow stromal precursors, is often combined with CD146 or CD271 to improve sorting efficiency (Janel et al., 2017; Miteva et al., 2025; Pearl et al., 2025). Recent studies have identified CD201 (PROCR) as a surface marker that inversely correlates with osteogenic potential, with CD201^Low^ adventitial progenitor cells displaying superior osteogenic differentiation and *in vivo* bone formation compared to their CD201^High^ counterparts (Wang et al., 2024a). From a developmental perspective, osteogenesis and chondrogenesis share a bipotent osteochondral progenitor stage governed by reciprocally antagonistic and synergistic transcriptional networks. Classical markers, however, can enrich these bipotent progenitors but lack the resolution to distinguish subpopulations biased toward either lineage. Recently, novel functional markers have provided more refined tools to isolate lineage-biased subsets from heterogeneous MSCs. This section focuses on these emerging markers, outlining their selection mechanisms and research progress in bone and cartilage regeneration. We also summarized the functional markers in this section in tables (Table 1).

**TABLE 1:** MSC functional markers for osteogenesis, chondrogenesis, and adipogenesis and their mechanisms.

Function	Marker	Cell source	Validated Species	Core function and mechanism	Detection condition	References
Osteogenesis and Chondrogenesis	CMKLR1^+^	Bone marrow MSCs	Human	Enhanced osteogenic differentiation and immunomodulatory capacity, attenuated adipogenic differentiation	In vitro-induced	(Xie et al., 2022)
	LIFR^+^/PDGFRβ^+^	Fetal bone marrow MSCs	Human fetal	Fetal-specific MSC marker, forms skeletal tissue *in vivo* and effectively reconstructs hematopoietic microenvironment	*In vivo*/*in situ*	(Zhang, P. et al., 2023)
	Kremen1^+^	Aged bone marrow MSCs	Rat	Specific surface marker of senescent MSCs; high Kremen1 expression is closely associated with decreased osteogenic differentiation capacity	In vitro-induced	(Wang et al., 2024b)
	CD34^+^/THY1^+^	Synovial fibroblasts with MSC characteristics	Human	Double-positive subpopulation exhibits the strongest osteogenic and chondrogenic capacity; THY1 knockdown inhibits osteogenic differentiation	*In vivo*/*in situ*	(Noda et al., 2022)
Adipogenesis	CD49f^+^	Adipose MSCs	Mouse and Rat	Functional marker of ADSCs with bidirectional enhancement of osteogenesis/adipogenesis	In vitro-induced	(Zha et al., 2021a)
	CD49f^+^/CD146^+^	Bone marrow and tonsil-derived MSCs	Human	Combinatorial marker screening strategy; osteogenic/adipogenic potential superior to single markers	In vitro-induced	(Tran et al., 2023)
	CD36^+^/MSCA1^+^	Pediatric adipose progenitor cells	Human	Marker of functional adipose progenitor subtypes; early association with obesity-related metabolic alterations validated in children	*In vivo*/*in situ*	(Hanschkow et al., 2022)
	CD10^+^	Adipose MSCs	Human	ADSCs overexpressing CD10 exhibit superior adipogenic capacity; inhibits lipolysis and promotes lipid accumulation through non-PPARγ-dependent mechanisms, positively correlated with browning potential	In vitro-induced	(Chakraborty et al., 2021)
	BAMBI^high^/MFGE8^high^	Umbilical cord MSCs	Human	Sorted subpopulation exhibits impaired adipogenic differentiation and weakened immunosuppressive function, representing a dysfunctional subpopulation within mixed MSC populations	In vitro-induced	(Chen et al., 2022)

#### 3.1.1 CMKLR1^+^

CMKLR1 (ChemR23), a G-protein coupled receptor, regulates the osteogenic–adipogenic balance in MSCs(Zhao et al., 2019; Guo et al., 2020a). Using single-cell transcriptomic sequencing, Xie et al. classified human bone marrow MSCs into CD26^+^ stemness, CMKLR1^+^ functional, and proliferative subsets. The CMKLR1^+^ subset exhibited markedly enhanced osteogenic differentiation and immunomodulatory capacity. Importantly, the proportion of CMKLR1^+^ cells increased from 37.4% at passage 1%–62.5% at passage 3, mirroring the stronger osteogenic ability of later-passage MSCs. This indicates that functional potency depends more on the proportion of this subset than on passage number *per se* (Xie et al., 2022). CMKLR1 therefore serves as a functional surface marker for enriching highly potent MSC subpopulations, offering a new strategy to improve therapeutic efficacy.

#### 3.1.2 LIFR^+^/PDGFRβ^+^

Leukemia inhibitory factor receptor (LIFR) and platelet-derived growth factor receptor β (PDGFRβ) are transmembrane receptors involved in stemness maintenance and osteogenesis, respectively (Chen et al., 2015; Ye et al., 2016; Wu et al., 2021). Through single-cell sequencing and lineage tracing, Zhang et al. demonstrated that the LIFR^+^/PDGFRβ^+^ double-positive subset in fetal bone marrow possesses the capacity to differentiate into osteoblasts *in vivo*, with osteogenic potential significantly surpassing that of other subsets, while also supporting the hematopoietic microenvironment. This marker combination is specific to fetal MSCs and is expressed at very low levels in adult MSCs, indicating an early developmental stage with strong bone-forming potential. Moreover, *in vitro* culture led to loss of LIFR expression and concurrent upregulation of classical markers such as CD73, CD90, and CD105, underscoring that culture itself remodels the MSC surface profile (Zhang, P. et al., 2023). LIFR^+^/PDGFRβ^+^ thus overcomes the limitation of conventional markers in distinguishing functional subsets and developmental stage heterogeneity, enabling the selection of MSCs with high osteogenic capacity from a developmental perspective.

#### 3.1.3 Kremen1^+^

In contrast to the fetal marker combination described above, aging MSCs gradually lose osteogenic ability and express distinct surface markers. Kremen1, a transmembrane antagonist of Wnt signaling, negatively regulates the canonical Wnt/β-catenin pathway by forming a complex with Dkk1 that blocks LRP5/6 receptors (Nakamura et al., 2008). Wang et al. identified Kremen1 as a specific surface marker of senescent bone marrow MSCs; the positive fraction rose to 75% in aged cells and was localized to the cell membrane. High Kremen1 expression closely correlates with diminished osteogenic differentiation. Based on this, the researchers constructed an anti-Kremen1 antibody-targeted nanoplatform to deliver a SIRT1 activator and NAD^+^ precursor specifically to senescent MSCs, successfully restoring their osteogenic capacity (Wang et al., 2024b). Kremen1 thus marks the senescent state of MSCs and can be used to select young cells that retain osteogenic ability or to assess MSC quality for bone tissue engineering. Notably, while KREMEN1 is known as a transmembrane protein involved in Wnt/β-catenin signaling modulation and cell survival in humans, its validation as a specific senescence marker has thus far been confined to the rat system, and its expression and functional relevance in human MSCs remain to be established.

#### 3.1.4 CD34^+^/THY1^+^

CD34, a classical hematopoietic stromal and progenitor cell marker (Molgaard et al., 1989), and THY1 (CD90), widely expressed on MSCs and fibroblasts (Moraes et al., 2016; Zeng et al., 2023), are co-expressed on a subset of synovial MSCs. Notably, the CD34^+^/THY1^+^ marker combination is not restricted to synovial tissue. It also identifies a subpopulation within adipose-derived stromal cells (ADSCs), which are generally characterized as CD34^+^/CD31^-^(Lin et al., 2010). In the synovial context, Noda et al. isolated three subsets from arthritic synovial tissue and demonstrated that CD34^+^/THY1^+^ double-positive cells constitute a synovial MSC population with high expression of typical MSC markers such as CD73 and CD271. Their osteogenic and chondrogenic capacities were significantly superior to those of CD34^-^/THY1^+^ and CD34^-^/THY1^-^ subsets, with no difference in adipogenic ability among the groups. THY1 knockdown further confirmed the functional necessity of this molecule for osteogenesis. Notably, CD34 is present on freshly isolated cells but rapidly lost during *in vitro* expansion, indicating it is a transient marker reflecting the cell’s original state within the tissue niche (Noda et al., 2022). This study highlights the utility of the CD34^+^/THY1^+^ dual-positive strategy for enriching synovial osteochondral progenitors and provides an important example of dynamic marker expression.

### 3.2 Adipogenesis

Adipogenic differentiation is a hallmark of MSC multipotency and is tightly regulated by a cascade of transcriptional events culminating in lipid accumulation. The capacity for adipogenesis not only reflects MSC functionality but also carries clinical implications for reconstructive surgery, metabolic disease modeling, and cell therapy quality control (Choi and Lee, 2020; Aabling et al., 2025). Among the classical surface markers, CD10 and CD36 have been consistently associated with adipogenic competence. CD10, originally identified as a specific marker of subcutaneous adipose MSCs, shows a positive correlation between its expression level and adipogenic differentiation efficiency (Ong et al., 2014). CD36, a fatty acid translocase, mediates fatty acid uptake and serves as a predictor of high adipogenic potential (Gao et al., 2017). However, MSCs from different tissues exhibit marked differences in adipogenic capacity, and even within a single tissue, clonal adipogenic potential varies considerably. Moreover, adipogenesis shares an early mesenchymal progenitor stage with osteogenesis and chondrogenesis, with a reciprocal balance among the three lineages, yet classical single markers cannot adequately resolve these functionally distinct subsets (Chen et al., 2016). In recent years, dual-positive strategies such as CD49f+/CD146+ and CD36+/MSCA1+, together with the functional re-evaluation of CD10, have substantially advanced the precision of adipogenic subset identification. This section focuses on these markers, their selection mechanisms, and their applications in adipogenic differentiation and tissue engineering (Table 1).

#### 3.2.1 CD49f^+^/CD146^+^

CD49f-positive MSCs are considered a primitive, stemness-related population with self-renewal and proliferative capacity; when combined with CD146, they help define early stromal precursors. Importantly, CD49f expression is not restricted to adipogenic progenitors, as CD49f^+^ MSCs also exhibit robust osteogenic potential (Tran et al., 2023). Zha et al. showed that magnetically sorted CD49f^+^ mouse/rat adipose-derived mesenchymal stromal cells (ADSCs) exhibit markedly enhanced proliferation, osteogenesis, adipogenesis, migration, and anti-apoptotic ability (Zha et al., 2021a). CD146 is also intimately linked to MSC function. Miteva et al. demonstrated that CD146^+^ stromal cells from human exfoliated deciduous teeth display significantly better osteogenic and adipogenic capacity than CD146^-^ cells (Zha et al., 2021a). Nguyen-Tran et al. further revealed that CD49f and CD146 are synergistically expressed, and the CD49f^+^/CD146^+^ double-positive subpopulation exhibits the strongest osteogenic and adipogenic capacity in both bone marrow mesenchymal stromal cells (BMSCs) and tonsil-derived mesenchymal stromal cells (TMSCs), whereas single-positive subsets show markedly attenuated function (Tran et al., 2023). Notably, responses to differentiation stimuli are tissue-specific. CD49f expression is significantly downregulated upon adipogenesis in TMSCs but maintained in BMSCs, highlighting the need to consider tissue origin when applying functional markers.

#### 3.2.2 CD36^+^/MSCA1^+^

CD36 (fatty acid translocase, FAT) and MSCA1 (tissue-nonspecific alkaline phosphatase, ALPL) are well-established surface markers associated with adipogenesis. CD36 mediates fatty acid uptake, and its expression correlates positively with adipogenic efficiency (Gao et al., 2017). MSCA1 enzymatic activity is required for triglyceride accumulation, and the MSCA1^+^ subset possesses the highest adipogenic potential with beige adipocyte precursor features (Estève et al., 2015). Hanschkow et al. demonstrated for the first time in children that the MSCA1^+^/CD36^+^ double-positive subpopulation represents the most adipogenic adipose progenitor functional subtype. MSCA1 expression positively correlates with adipocyte hypertrophy, mitochondrial respiration, and systemic inflammatory markers, while CD36 expression correlates with adipogenic efficiency and is significantly downregulated in obese children (Hanschkow et al., 2022). Notably, although MSCA1 was originally an MSC marker, the MSCA1^+^/CD36^+^ cells in this study are actually adipose progenitors with restricted multipotency but high adipogenic efficiency, rather than classical MSCs. This marker combination thus labels a functional subset along the MSC-to-adipocyte differentiation trajectory.

#### 3.2.3 CD10^+^

CD10 was originally identified as a marker highly expressed in subcutaneous adipose MSCs, with its expression positively correlated with adipogenic capacity (Ong et al., 2014). However, subsequent studies have revealed that CD10 is not restricted to the adipogenic lineage but is also linked to osteogenesis (Ding et al., 2020). Chakraborty et al. recently demonstrated through knockdown and overexpression experiments that CD10 not only serves as a passive marker but directly participates in adipogenic regulation. The study revealed that CD10 promotes lipid accumulation through a PPARγ-independent mechanism, specifically by inhibiting the phosphorylation of key lipolytic enzymes, hormone-sensitive lipase and adipose triglyceride lipase, thereby reducing triglyceride breakdown. Moreover, the study proved that ADSCs with high CD10 expression showed a more pronounced upregulation of uncoupling protein-1 upon forskolin stimulation, linking CD10 to browning potential for the first time (Chakraborty et al., 2021). These findings elevate CD10 from a predictive indicator to a functional regulatory molecule, offering a mechanism-driven strategy for selecting high-quality adipose MSCs.

### 3.3 Angiogenesis

Angiogenesis, the formation of new blood vessels from pre-existing vasculature, is a core process in tissue repair and functional reconstruction. MSCs have emerged as promising therapeutic agents for ischemic diseases owing to their multilineage potential and robust paracrine properties, particularly their ability to secrete an array of pro-angiogenic factors. Classical surface markers such as CD146, PDGFRβ, PDGFRα, NG2, α-SMA, and Nestin have long served as key identifiers of pro-angiogenic MSC subsets. CD146 mediates pericyte adhesion and vascular stabilization, PDGFRβ and PDGFRα regulate pericyte recruitment and vessel maturation, and NG2, α-SMA, and Nestin mark contractile perivascular subpopulations (Rasile et al., 2022; Zhu et al., 2022; Cao et al., 2023). CD248, an emerging pericyte marker, further aids in identifying perivascular subsets missed by traditional markers (Naylor et al., 2014). Despite the utility of these markers in isolating perivascular MSC populations, they do not uniformly predict pro-angiogenic potency. The same marker may denote cells with divergent angiogenic capacity depending on tissue origin, and the paracrine output of functionally distinct subsets cannot be distinguished by classical markers alone. Recent studies have therefore expanded the repertoire of angiogenic MSC markers to include tissue-specific regulators such as ABCB5, as well as new functional applications of CD146 in endometrial MSCs and CD271 in adipose-derived MSCs. This section examines these emerging markers and their contributions to pro-angiogenic MSC therapy. A summary of the functional markers in this section is provided in the tables (Table 2).

**TABLE 2:** MSC functional markers for angiogenesis, neurogenesis, myogenesis, and epitheliogenesis and their mechanisms.

Function	Marker	Cell source	Validated Species	Core function and mechanism	Detection condition	References
Angiogenesis	CD146^+^	Endometrial MSCs	Human	Highly secreted VEGF and TGF-α, effectively promoting endothelial-like structure formation and chick embryo angiogenesis	In vitro-induced	(Hilage et al., 2024)
	CD271^+^	Adipose MSCs	Human	Enriched pro-angiogenic subpopulation in adipose MSCs	*In vivo*/*in situ*	(Smith et al., 2021)
	ABCB5^+^	Dermal MSCs	Human	Promoted angiogenesis through HIF-1α/VEGF pathway and SERCA2a-PLN axis	*In vivo*/*in* *situ*	(Kerstan et al., 2022; Yan et al., 2023)
Neurogenesis	GD2^+^	Umbilical cord blood-derived MSCs	Human	Identified MSC subpopulation with neural differentiation potential; predicted neural induction efficiency	In vitro-induced	(Jin et al., 2010)
	CD271^+^/CD56^+^	Bone marrow MSCs	Human	Sorted and extracted exosomes promoted axonal growth through miR-431-3p/RGMA axis	*In vivo*/*in situ*	(Sun et al., 2024)
	SSEA3^+^/CD105^+^ (Muse cells)	Nasal turbinate stem cells (NTSCs)	Human	Sorted cells highly expressed BDNF, GDNF, etc., effectively protecting nerves and promoting synapse formation	In vitro-induced	(Lim et al., 2026)
Myogenesis	CD56^+^	Skeletal muscle-derived MSCs	Human	Significantly higher myogenic differentiation potential, with stronger response to non-cytotoxic heat stimulation (42 °C, 1h)	*In vivo*/*in situ*	(Mikšiūnas et al., 2022)
	CD271^+^	Bone marrow MSCs	Dog	Stronger proliferative capacity; significantly enhanced myogenic function after MyoD adenoviral transduction	*In vivo*/*in situ*	(Nitahara-Kasahara et al., 2012)
Epithelial regeneration	CD34^+^	Intestinal mesenchymal stromal cells	Mouse	Maintained intestinal epithelial stem cell stemness and promoted epithelial regeneration and repair by upregulating niche factors and inflammatory repair factors	*In vivo*/*in situ*	(Stzepourginski et al., 2017)
	CD146^+^/PDGFRβ^+^	Endometrial MSCs	Human	Promoted angiogenesis through paracrine VEGF-α, as well as anti-inflammatory and anti-fibrotic effects, indirectly promoting CK19^+^ epithelial cell proliferation and re-epithelialization	In vitro-induced	(Li et al., 2024)

#### 3.3.1 CD146^+^

CD146^+^ cells, originating from the perivascular niche, are classical functional MSC subsets with multilineage potential and enhanced immunosuppressive activity relative to CD146^-^ cells (Crisan et al., 2008); they contribute to blood vessel formation through paracrine actions and differentiation into vascular-associated cells (Sacchetti et al., 2007; Pearl et al., 2025). Although their pro-angiogenic role is well recognized, validation in endometrial stromal cells had been lacking. Hilage et al. isolated CD146^+^ cells from human endometrium for the first time and systematically demonstrated their strong pro-angiogenic capacity. These cells secrete high levels of VEGF and transforming growth factor α, rapidly form stable endothelial-like structures in Matrigel, and significantly increase vascular nodes and branches in a chick embryo model (Hilage et al., 2024). This study provides a novel cell source for treating ischemic vascular diseases.

#### 3.3.2 CD271^+^

CD271 (p75 neurotrophin receptor, p75NTR) is widely recognized as a marker for enriching MSC subsets with high clonogenicity, trilineage differentiation, and immunomodulatory properties (Kuçi et al., 2010; Pearl et al., 2025). However, direct functional evidence for CD271 in angiogenesis had been relatively limited. Smith et al. enriched CD271^+^ ADSCs via magnetic bead sorting and found that this subset highly expresses angiogenic genes such as angiopoietin-2 and HGF, lowly expresses inflammatory genes such as interleukin-1 and interleukin-1β, and contains a higher proportion of CD90^+^/CD45^-^ stromal cells. In human umbilical vein endothelial cell co-culture, CD271^+^ ADSCs promoted the formation of more complex vascular networks, with increased tube branches and anastomotic points; upon co-culture with adipose tissue, angiopoietin-1 expression and secretion were markedly elevated (Smith et al., 2021). Collectively, CD271^+^ ADSCs possess dual pro-angiogenic and anti-inflammatory properties, making them potentially valuable in tissue engineering applications requiring revascularization, such as fat grafting.

#### 3.3.3 ABCB5^+^

ABCB5 was initially identified as a functional marker of skin MSCs, with the subset exhibiting immunomodulatory and trilineage differentiation capacities (Schatton et al., 2015). In terms of angiogenesis, ABCB5^+^ MSCs display clear pro-angiogenic ability. Under hypoxic conditions they stabilize hypoxia-inducible factor 1-α, upregulate VEGF, and can form capillary-like structures on Matrigel (Kerstan et al., 2022). Moreover, ABCB5^+^ MSCs indirectly protect angiogenic function by restoring calcium homeostasis in endothelial cells under hypoxia. Notably, the angiogenic effect of ABCB5^+^ MSCs is tissue-specific (Yan et al., 2023). Skin-derived ABCB5^+^ MSCs exert pro-angiogenic effects, whereas limbal-derived ABCB5^+^ MSCs exhibit anti-angiogenic properties due to lower secretion of VEGFA and VEGFC(Meshko et al., 2024). These findings indicate that the pro-angiogenic function of ABCB5^+^ MSCs is tissue-specific and influenced by culture conditions such as hypoxia, emphasizing that marker interpretation must account for cell source and microenvironmental factors, thereby guiding precise selection of MSC subsets for different indications.

### 3.4 Neurogenesis

Although MSCs are of mesodermal origin, they possess the capacity to differentiate toward neural lineages, a property that has attracted considerable interest for neurological repair (Table 2). Surface markers provide a means to identify MSC subsets with neurogenic potential and have been instrumental in advancing this field. Classical markers such as CD146, CD24, Stro-1, and CD44 are highly expressed in dental pulp MSCs and frequently co-localize with neurogenic markers like nestin and βIII-tubulin, indicating a neural crest-associated identity (Wang et al., 2024c; Roato et al., 2025; Chirico et al., 2026). GD2, a ganglioside surface antigen, was the first single marker shown to uniquely distinguish MSCs from other bone marrow elements and has been demonstrated to drive early neuronal differentiation through regulation of the bHLH transcription factor family (Jin et al., 2010). CD271, a low-affinity neurotrophin receptor, enriches neural crest-derived MSC populations with enhanced neurotrophic factor secretion (Saulite et al., 2018; Zha et al., 2021b), while CD56 marks subsets with intrinsic neurogenic bias (Kim et al., 2014). Collectively, these classical markers have established the foundation for isolating MSC subpopulations with neural lineage potential.

Recent advances have moved beyond single-marker identification toward multi-marker strategies and functional characterization of paracrine mechanisms. Exosomes derived from CD271^+^/CD56^+^ double-positive bone marrow MSCs significantly enhance axon outgrowth and branching of dorsal root ganglion neurons through the miR-431-3p/RGMA axis (Sun et al., 2024). SSEA3^+^/CD105^+^ Muse cells exhibit strong neuroprotective effects by secreting BDNF and GDNF, reducing neuronal apoptosis and promoting synaptic protein expression (Lim et al., 2026). These findings indicate a paradigm shift from using surface markers as static identity labels to employing them as tools for capturing dynamic functional states, including paracrine neurotrophic support. Future research is expected to integrate single-cell multi-omics with high-parameter flow cytometry to discover novel marker combinations that can discriminate between direct neuronal differentiation and indirect neurotrophic mechanisms, thereby enabling precise selection of MSC subsets tailored to specific neurological indications.

### 3.5 Myogenesis

MSCs contribute to muscle development, homeostasis maintenance, and injury repair through both direct myogenic differentiation and paracrine support of resident satellite cells (Table 2). The identification of surface markers capable of distinguishing myogenic MSC subsets is therefore critical for developing cell-based therapies for muscular disorders. Functional surface markers such as CD56 have been successfully used to enrich satellite cell-like subsets from human skeletal muscle; these CD56^+^ cells exhibit enhanced expression of late myogenic markers including myogenin, creatine kinase, and myosin heavy chain, as well as robust myotube formation (Scott et al., 2013; Xu et al., 2015; Mikšiūnas et al., 2022). CD271^+^ bone marrow MSCs display greater proliferative potential and, upon MyoD transduction, undergo efficient myogenic differentiation through the classical myogenic transcriptional cascade, forming myotubes and even muscle-like tissue *in vivo* (Nitahara-Kasahara et al., 2012). Unlike the above-mentioned studies on CD271, the present work is restricted to canine models, providing key information for DMD preclinical research in dogs. These results cannot be directly extrapolated to humans, but they offer solid animal-model validation for future clinical translation of this strategy. Additionally, CD146^+^ perivascular cells and PDGFRβ^+^ populations have been implicated in muscle regeneration, demonstrating the value of these markers in isolating cells with myogenic capacity (Contreras et al., 2021; Lu et al., 2022).

Recent investigations have revealed that not all marker-defined populations represent *bona fide* multipotent MSCs. Skeletal muscle CD146^+^ pericytes, despite expressing classical ISCT-defined MSC markers, have been shown to be committed myogenic progenitors rather than traditional multipotent MSCs(Sacchetti et al., 2016). This finding underscores the limitation of using conventional surface markers alone to distinguish between true MSCs and lineage-restricted progenitors within muscle tissue. Ongoing research aims to identify more specific marker combinations that can reliably separate multipotent MSCs from committed myogenic cells. Such advances will be essential for developing standardized, predictable MSC-based therapies for muscle diseases.

### 3.6 Epitheliogenesis

Epithelial tissues, including the intestinal mucosa, epidermis, and endometrium, depend on resident stromal cell pools for continual renewal and post-injury repair (Table 2). MSCs support epithelial regeneration primarily through paracrine mechanisms rather than direct differentiation, serving as niche-supporting cells that secrete trophic factors and modulate the local regenerative microenvironment. Classical surface markers have been instrumental in isolating MSC subsets with epithelial support capacity. In the mouse intestine, CD34^+^ stromal cells maintain Lgr5+ intestinal epithelial stem cells through secretion of Wnt2b, R-spondin1, and Gremlin1, which is essential for epithelial regeneration after injury (Stzepourginski et al., 2017; Ayansola et al., 2024). In the endometrium, PDGFRβ^+^/CD146^+^ and SUSD2^+^ subsets identify perivascular MSC populations capable of supporting endometrial epithelial repair (Schwab and Gargett, 2007; Masuda et al., 2012). These classical markers have thus enabled the enrichment of MSCs that promote epithelial healing through niche-dependent mechanisms.

Current research on novel functional markers remains focused on the paracrine support paradigm. Menstrual-derived stromal cells and endometrial MSCs have been shown to facilitate epithelial repair by secreting pro-angiogenic and anti-inflammatory factors, yet no surface marker has been identified that can isolate MSCs capable of direct epithelial differentiation. Some studies have reported that mouse CD34^+^/KLF4^+^ stromal cells undergo mesenchymal-epithelial transition to form E-cadherin^+^ epithelial structures (Yin et al., 2019), but such direct transdifferentiation has not been confirmed in human tissues. Future directions include leveraging single-cell multi-omics to search for early surface signatures of epithelial commitment within MSC populations, and developing functional markers that specifically identify subsets with enhanced paracrine activity tailored to distinct epithelial niches. Such advances could expand the therapeutic utility of MSCs in epithelial regeneration beyond their current niche-supporting role.

## 4 Application of functional marker-sorted MSC subpopulations in disease models and clinical therapy

Harnessing functional surface markers to identify and enrich MSC subpopulations with defined therapeutic properties is a core strategy for precision cell therapy. This approach has demonstrated substantial value across a wide spectrum of preclinical models and clinical investigations, spanning neurological, cardiovascular, cutaneous, musculoskeletal, and hepatobiliary diseases. This chapter systematically reviews the application of functionally distinct MSC subsets in these disease categories.

### 4.1 Neurological disorders

Neurological disorders such as spinal cord injury (SCI), stroke, Alzheimer’s disease, and drug-resistant epilepsy in children, despite differing etiologies, share the common pathological features of blood-central nervous system barrier disruption, capillary endothelial damage, and sustained neuroinflammation (Acioglu and Elkabes, 2025; Lemarchant et al., 2025; Xiang et al., 2025). Given the marked functional heterogeneity of conventional MSC preparations, identifying functional surface markers that can prospectively isolate subpopulations with superior neural repair capacity has become a pivotal step toward precision therapy for these conditions. We summarized the recent application of functional marker-sorted MSC subpopulations in neurological disorders in tables (Table 3).

**TABLE 3:** Recent applications of functional marker-sorted MSC subpopulations in neurological and circulatory system disorders.

Disorders	Indication	Marker	Cell source	Outcomes	References
Neurological Disorders	Stroke	CD133^+^	Bone marrow MSCs	Intra-arterial injection of concentrated CD133^+^ BMSC-conditioned medium provided neuroprotective effects in mice and significantly reduced cortical infarct volume after cerebral ischemia	(Bakondi et al., 2009)
	Spinal Cord Injury	CD271^+^/CD56^+^	Bone marrow MSCs	In SCI models, *in situ* implantation of CD271^+^/CD56^+^ BMSC-Exos hydrogel promoted axonal regeneration and synapse formation, contributing to neurological functional recovery	(Sun et al., 2024)
	​	CD146^+^/CD271^+^	Umbilical cord MSCs	In SCI models, intranasal administration of RGD-CD146^+^/CD271^+^ umbilical cord MSC exosomes could target neovascular endothelial cells, attenuate blood-spinal cord barrier disruption, and improve neurological functional recovery	(Xie et al., 2023)
	Alzheimer’s Disease	SSEA3^+^/CD105^+^	NTSCs	Both higher-ratio and purified SSEA3^+^/CD105^+^ NTSCs reduced brain Aβ deposition, inflammation, and neuronal death in AD model mice, improving cognitive function	(Lim et al., 2026)
	Drug-Resistant Epilepsy in Children	CD271^+^	Bone marrow MSCs	Follow-up over 2–4 years showed that autologous transplantation was well tolerated and reduced seizure frequency and status epilepticus, improving neurocognitive function (e.g., cognition, emotion, motor ability)	(Milczarek et al., 2018; Milczarek et al., 2024)
Circulatory System Disorders	Myocardial Infarction	CD73^+^	Adipose MSCs	Promoted angiogenesis in rat myocardial infarction models and accelerated cardiac functional recovery	(Li, Q. et al., 2021)
	​	CD51^+^	Bone marrow MSCs	After intravenous transplantation into mice with myocardial infarction, more effectively migrated to and engrafted in the infarcted heart, thereby better improving cardiac function and partially attenuating inflammatory responses	(Xie, D. M. et al., 2019)
	​	CD51^+^	Cardiac MSCs	Promoted angiogenesis through secretion of stem cell factors, significantly improving cardiac function and attenuating cardiac fibrosis in mice with myocardial infarction	(Xie M. et al., 2021)
	Peripheral Arterial Disease	CD141^+^	Vascular precursor cells	Human CD141^+^ VPCs and BMSCs were co-transplanted intramuscularly at a 2:1 ratio in a critical limb ischemia model with femoral artery ligation, achieving limb salvage, blood flow restoration, and large-vessel regeneration, with efficacy superior to single transplantation	(Park et al., 2024)
	​	ABCB5^+^	Dermal MSCs	In a mouse acute-on-chronic ischemia model, increased microvessel density and attenuated muscle injury	(Yan et al., 2023)

#### 4.1.1 Stroke

Following ischemic or hemorrhagic stroke, blood-brain barrier breakdown and the ensuing secondary injury are central to neurological deterioration (Wang et al., 2025; Chen et al., 2026), making barrier restoration and inflammation suppression key therapeutic goals. Surface marker-based enrichment of MSC subsets with potent anti-inflammatory and vascular repair functions can significantly enhance the precision and efficacy of post-stroke neural repair. A study has shown that the conditioned medium of CD133^+^ bone marrow MSCs, owing to their robust paracrine activity, substantially reduces cortical infarct volume and confers neuroprotection in murine ischemic stroke models (Bakondi et al., 2009).

#### 4.1.2 Spinal cord injury (SCI)

Spinal cord injury disrupts the blood-spinal cord barrier, triggering massive infiltration of neutrophils and macrophages and leading to irreversible neurological deficits (Lentilhas-Graça et al., 2024). Consequently, MSC subsets capable of barrier repair and axonal regeneration are of particular therapeutic interest. Sun et al. identified a CD271^+^/CD56^+^ double-positive subset from human bone marrow mononuclear cells via single-cell sequencing; this subset is highly enriched in axonal development and neural regeneration pathways. Exosomes isolated from this subpopulation, loaded into a hydrogel and transplanted into a complete spinal cord transection model, significantly promoted axonal regeneration and motor recovery through the miR-431-3p/RGMA axis (Sun et al., 2024). Moreover, synergistic sorting with CD146 and CD271 has shown distinct advantages. Xie et al. isolated CD146^+^/CD271^+^ umbilical cord MSCs, modified their exosomes with RGD peptides, and delivered them intranasally to spinal cord-injured mice. The exosomes specifically accumulated in the lesion site, reduced vascular leakage, upregulated tight junction proteins, repaired the blood-spinal cord barrier, and facilitated neurological recovery (Xie et al., 2023).

#### 4.1.3 Alzheimer’s disease (AD)

Alzheimer’s disease is characterized by β-amyloid deposition, tau hyperphosphorylation, chronic neuroinflammation, and blood-brain barrier dysfunction (Chen, X. et al., 2023). An effective stromal cell therapy must address multiple targets, including crossing the blood-brain barrier, delivering neurotrophic factors, and modulating microglial activity. Lim et al. applied the SSEA3^+^/CD105^+^ Muse cell sorting strategy to Alzheimer’s disease treatment. Using human nasal turbinate neural crest-derived stromal cells, they demonstrated that the proportion of Muse cells positively correlated with cognitive improvement, Aβ plaque reduction, and microglial suppression in 5×FAD transgenic mice. Importantly, a high ratio of SSEA3/CD105-positive cells without further purification was sufficient to achieve equivalent therapeutic benefits, and these cells restored calcium signaling oscillations and synaptic protein colocalization in brain organoids derived from Alzheimer’s patient iPSCs(Lim et al., 2026). This finding provides a simple and cost-effective strategy for MSC product quality control in clinical settings.

#### 4.1.4 Drug-resistant epilepsy in children

Drug-resistant epilepsy in children is characterized by recurrent aberrant neuronal discharges that are refractory to conventional antiepileptic drugs, which also carry risks of neurodevelopmental toxicity (Kwan et al., 2010; Lolam and Roy, 2025). Neuroinflammation is a key driver of epileptogenesis, and MSCs can repair damaged neural networks by secreting anti-inflammatory cytokines and neurotrophic factors. CD271 plays a critical role in neural development and regeneration. Milczarek et al. conducted a clinical study of autologous CD271^+^ bone marrow MSC transplantation in children with drug-resistant epilepsy. Over 2–4 years of follow-up, seizure frequency dropped from dozens per week to 1–7 per week, status epilepticus episodes ceased, EEG discharges decreased, and cognitive and motor functions improved, with no serious adverse events and sustained efficacy after treatment cessation (Milczarek et al., 2018; Milczarek et al., 2024). Mechanistically, CD271^+^ MSCs highly express BDNF, GDNF, VEGF, and other neurotrophic factors and possess immunomodulatory potential.

### 4.2 Circulatory system disorders

Cardiovascular and peripheral vascular diseases are fundamentally characterized by vascular network destruction and inadequate tissue perfusion, making angiogenesis a critical therapeutic target (Chen, X. et al., 2023). MSCs promote ischemic tissue repair by secreting pro-angiogenic factors and differentiating into vascular endothelial cells. However, unsorted MSCs exhibit limited angiogenic potency and substantial inter-subset variability. In recent years, sorting MSC subpopulations with high pro-angiogenic capacity using functional surface markers has yielded breakthroughs in multiple ischemic disease models (Table 3).

#### 4.2.1 Myocardial infarction

After myocardial infarction, coronary occlusion leads to massive cardiomyocyte death and reduced vascular density in the infarct zone; thus, promoting angiogenesis to restore perfusion is essential for improving cardiac function (Cahill et al., 2017; Qian and Yao, 2026). MSC heterogeneity results in inconsistent therapeutic outcomes. Li et al. obtained CD73^+^ adipose-derived MSCs by flow cytometry and found that they secreted significantly more VEGF, SDF-1α, and HGF than CD73^−^ cells. Transplantation of CD73^+^ cells markedly increased vascular density in the infarct region of rats, improved left ventricular ejection fraction by approximately 15%, and significantly reduced left ventricular end-systolic volume (Li, Q. et al., 2021). Furthermore, CD51 exemplifies the dynamic regulation of surface markers between normal and diseased states. Xie et al. reported that CD51 expression in the murine heart is abundant in neonatal stages but declines with age, while its local expression increases upon myocardial injury, suggesting an injury-responsive program. Functionally, Xie et al. reported that CD51^+^ bone marrow MSCs exhibit enhanced homing to the infarct zone via high chemokine receptor expression, while cardiac-derived CD51^+^ MSCs promote angiogenesis through stem cell factor secretion (Xie et al., 2019; Xie, D. M. et al., 2021). Collectively, these findings illustrate that markers such as CD51 not only enable the enrichment of therapeutically relevant MSC subsets but also undergo dynamic changes in response to pathological cues.

#### 4.2.2 Peripheral arterial disease

Peripheral arterial disease, particularly critical limb ischemia, involves severe distal hypoperfusion with a risk of amputation; thus, rebuilding functional vascular networks is the primary therapeutic goal (Norgren et al., 2007). Functional subset-based cell therapy offers a novel approach. CD141, a classical endothelial marker, identifies vascular precursor cells possessing both MSC and endothelial characteristics (Loghmani and Conway, 2018). Park et al. co-transplanted CD141^+^ vascular precursor cells (VPCs) with bone marrow MSCs at a 2:1 ratio into a nude mouse hindlimb ischemia model. Blood flow recovered to normal levels, limb salvage reached 90%, and the transplanted cells differentiated into CD31^+^ endothelial and αSMA^+^ vascular smooth muscle cells. This dual-cell therapy mimics the physiological endothelial-pericyte synergy and outperforms single-cell transplantation (Park et al., 2024). In addition, Yan et al. demonstrated that ABCB5^+^ dermal MSCs restore calcium homeostasis disrupted by hypoxia through upregulating SERCA2a expression and promoting PLN phosphorylation; intramuscular injection accelerated blood flow recovery, increased microvascular density, and reduced muscle injury (Yan et al., 2023).

### 4.3 Skin and mucosal disorders

Skin and mucosal wound healing involves inflammation resolution, cell proliferation and migration, angiogenesis, re-epithelialization, and matrix remodeling (Gonzalez et al., 2016). To precisely select MSC subpopulations capable of coordinating these reparative events, researchers have sought to identify corresponding functional surface markers.

#### 4.3.1 Skin wound repair

Robust angiogenesis is central to skin wound repair, and marker selection should focus on subsets with strong pro-angiogenic and immunomodulatory functions. Brett et al. identified CD248 as a key marker of a pro-angiogenic subpopulation in human adipose stromal vascular fraction cells through single-cell transcriptomics. CD248^+^ cells highly express VEGF and HGF and promote endothelial tube formation *in vitro*. In a mouse full-thickness skin defect model, wounds treated with CD248^+^ cells achieved complete closure by day 13, 2–3 days earlier than controls, and exhibited significantly higher neovascular density (Brett et al., 2017). Additionally, Rezzonico Jost et al. found that in autologous fat grafting for scar improvement, the proportion of CD304^+^ adipose-derived MSCs was positively correlated with long-term pain relief, a mechanism attributed to high CD10 expression that degrades the pain mediator bradykinin and high CD73 expression that generates the anti-inflammatory and analgesic molecule adenosine (Rezzonico Jost et al., 2024). We have summarized the recent application for this section in tabular form (Table 4).

**TABLE 4:** Recent applications of functional marker-sorted MSC subpopulations in skin and mucosal disorders, bone and joint disorders, and hepatobiliary disorders.

Disorders	Indication	Marker	Cell source	Outcomes	References
Skin and Mucosal Disorders	Skin Wound Repair	CD248^+^	Stromal vascular fraction cells	Treatment of full-thickness skin wounds in immunodeficient mice within a hydrogel significantly accelerated healing, promoted re-epithelialization, and increased neovascularization	(Brett et al., 2017)
	​	CD304^+^	Adipose MSCs	May synergistically enhance autologous fat graft-induced collagen deposition and neovascularization through paracrine signaling, thereby improving tissue quality and modulating local nociceptive signaling	(Rezzonico Jost et al., 2024)
	Endometrial Injury	PDGFRβ^+^/CD146^+^	Endometrial MSCs	In a highly immunodeficient mouse model of endometrial electrocautery injury, significantly promoted endometrial repair, regulated inflammation, reduced fibrosis, and improved embryo implantation rates	(Li et al., 2024)
Bone and Joint Disorders	Arthritic Diseases	CD47^high^	Synovial MSCs	Exhibited potent therapeutic effects in a rat osteoarthritis model and directly promoted new articular cartilage formation	(Al-Jezani et al., 2026)
	​	CD248^+^	Skin and synovial MSCs	Highly expressed on inflammatory MSCs; both membrane-bound and soluble CD248 may be involved in the pathogenesis of rheumatoid arthritis	(Payet et al., 2023)
	Bone and Cartilage Defects	CD146^+^	Adipose MSCs	In a mouse articular cartilage defect model, demonstrated stronger tissue repair capacity based on gross observation, histological observation, and histological scoring	(Xi et al., 2021)
	​	Kremen1^+^	Bone marrow MSCs	In aged rat bone defect models, a designed senescence-targeting platform increased new bone volume fraction and trabecular number, and significantly reduced p16^+^/LepR^+^ senescent MSC counts, promoting bone tissue regeneration	(Wang et al., 2024b)
	​	CD271^+^	Bone marrow MSCs	Significantly promoted long-term new bone formation in an *in vivo* rat femoral condyle defect model	(Sun et al., 2022)
Hepatobiliary Disorders	liver fibrosis/cirrhosis	NG2^+^	Bone marrow MSCs	In mouse models of liver fibrosis/cirrhosis, differentiated into biliary cells and generated hepatic sinusoidal endothelial cells to reconstruct vasculature and sinusoidal structures, initiating hepatocyte regeneration and promoting liver tissue repair and functional recovery	(Hu et al., 2024)
	​	NG2^+^	Bone marrow MSCs	In liver failure models, effectively reconstructed microvasculature and sinusoidal structures within decellularized liver scaffolds for constructing transplantable bioengineered livers, thereby prolonging survival time in partial hepatectomy pig models	(Lai et al., 2025)
	​	CD36^+^	Bone marrow MSCs	High CD36 expression in bone marrow MSCs from patients with alcoholic cirrhosis was associated with non-response after transplantation	(Gupta et al., 2019)

#### 4.3.2 Endometrial injury

Endometrial injury can cause intrauterine adhesions, menstrual abnormalities, and infertility, with the core pathology being stromal cell pool exhaustion and vascular destruction in the basal layer (Li, X. et al., 2021). Repair strategies that concurrently promote epithelial regeneration and revascularization are urgently needed. PDGFRβ and CD146 identify perivascular endometrial MSCs and can also be used to isolate subsets with endometrial repair and *in vitro* epithelial construction capacity. Li et al. transplanted human PDGFRβ^+^/CD146^+^ endometrial MSCs into a mouse model of endometrial electrocoagulation injury and intrauterine adhesions. The transplanted cells homed to the perivascular region, significantly enhanced proliferation, angiogenesis, epithelial regeneration, and stromal repair, ultimately improving endometrial thickness and gland formation, suppressing fibrosis, and restoring fertility (Li et al., 2024). More recently, Fang et al. identified a LEPR^+^ quiescent subpopulation within PDGFRβ^+^/CD146^+^ endometrial MSCs via single-cell sequencing that possesses enhanced stemness and clonogenicity, providing new targets for optimizing endometrial MSC-based regenerative therapy (Fang et al., 2025).

### 4.4 Bone and joint disorders

Bone and joint disorders, primarily including osteoarthritis and bone/cartilage defects, share the common pathological feature of progressive tissue destruction coupled with insufficient regenerative capacity (Swank et al., 2020; Zhang, Z. et al., 2024; Guo et al., 2026). MSC-based therapies hold promise, yet the heterogeneity of conventional MSC populations leads to inconsistent outcomes (Dickinson et al., 2017). In recent years, treatment strategies employing functional surface markers to sort specific MSC subsets have achieved notable progress in musculoskeletal disease models (Table 4).

#### 4.4.1 Arthritic diseases

Osteoarthritis is characterized by gradual cartilage degeneration and subchondral bone remodeling, with inadequate chondrogenic capacity being a key therapeutic bottleneck (Hu et al., 2021). Al-Jezani et al. identified CD47 as a functional marker of synovial MSC subsets through proteomic screening. CD47^hi^ cells exhibit superior *in vitro* chondrogenesis, can directly integrate into articular cartilage and differentiate into PRG4^+^/Sox9^+^ mature chondrocytes in a rat osteoarthritis model, significantly attenuating cartilage damage, with very low proliferative activity *in vivo*, indicating a direct differentiation-based contribution (Al-Jezani et al., 2026). This study provides the first evidence that functional marker-sorted MSC subsets can directly participate in cartilage regeneration *in vivo*. In the context of rheumatoid arthritis, inflammatory MSCs upregulate CD248, which promotes synovial inflammation and angiogenesis through both membrane-bound and soluble pathways. Targeting CD248^+^ inflammatory MSCs may therefore offer a new approach for modulating rheumatoid arthritis pathology, although their therapeutic application requires further exploration (Payet et al., 2023).

#### 4.4.2 Bone and cartilage defects

The core pathology of bone and cartilage defects is insufficient osteogenic/chondrogenic progenitor supply and limited *in situ* regeneration, making the precise selection of subsets with high differentiation potential essential for improving treatment outcomes (Deszcz and Bar, 2025). CD146 is considered an effective marker for identifying seed cells with high differentiation capacity in cartilage tissue engineering. Xie et al. used magnetic bead-sorted CD146^+^ adipose-derived MSCs, induced chondrogenesis *in vitro*, and transplanted them into a mouse articular cartilage defect model. The expression of cartilage markers such as Sox9, collagen II, and aggrecan was significantly higher than in unsorted ADSC controls, with superior histological scores and repair outcomes (Xie, A. et al., 2021). Diverging from strategies that enrich functionally enhanced subsets, another line of research targets senescent MSCs to restore their function. Wang et al. exploited the senescence-specific surface marker Kremen1 to construct a targeted nanoplatform co-delivering resveratrol and nicotinamide riboside. This platform activated the NAD^+^-dependent SIRT1 pathway, significantly delaying MSC senescence and restoring osteogenic capacity. In an aged rat bone defect model, this targeted platform markedly increased new bone volume fraction and reduced the number of p16^+^/LepR^+^ senescent MSCs, promoting bone regeneration (Wang et al., 2024b). Furthermore, CD271 has been innovatively applied for *in situ* bone regeneration. For example, CD271-antibody-functionalized microspheres successfully recruited endogenous CD271+ cells *in vivo*, significantly enhancing new bone formation (Sun et al., 2022).

### 4.5 Hepatobiliary disorders

End-stage liver disease is characterized by massive hepatocyte loss, hepatic sinusoidal vascular network destruction, and bile duct damage (Zhang, L. et al., 2024). The scarcity of donor organs limits liver transplantation, creating an urgent need for cell therapies capable of multifaceted reconstruction (Schuppan and Afdhal, 2008). A summary of the recent application in this section is provided in the table (Table 4). Neuron-glial antigen 2 (NG2) is upregulated in fibrotic livers of both humans and mice and can serve as a key marker to efficiently enrich MSC subpopulations with dual angiogenic and bile duct-forming potential (Yang et al., 2023). Hu et al. isolated bone marrow NG2^+^ cells and demonstrated that in a mouse model of liver fibrosis/cirrhosis, these cells differentiated into Lyve-1^+^ liver sinusoidal endothelial cells, reconstructed damaged hepatic sinusoids, and promoted hepatocyte regeneration, whereas unsorted bone marrow MSCs lacked this capacity (Hu et al., 2024). Building on these *in vivo* findings, Lai et al. further employed NG2^+^ bone marrow MSCs as “extrahepatic sinusoidal progenitors” seeded onto decellularized liver scaffolds. In a 3D bioreactor, they successfully constructed a bioartificial liver with vascular networks and bile duct structures. In a porcine model of approximately 90% hepatectomy, transplantation of this bioartificial liver significantly prolonged recipient survival, offering a novel transplantable strategy for end-stage liver disease (Lai et al., 2025). On a different note, aberrant CD36 expression can serve as a predictive marker for MSC therapy response. Gupta et al. found that high CD36 expression in bone marrow MSCs from patients with alcoholic cirrhosis was associated with non-response to MSC transplantation, suggesting that CD36 could be used as a negative selection marker to identify potential responders (Gupta et al., 2019). In summary, NG2 is an effective tool for enriching highly reparative MSC subsets, while CD36 provides a new molecular marker for predicting the efficacy of MSC transplantation.

## 5 Discussion and summary

Functional surface markers provide a crucial entry point for tackling MSC heterogeneity, yet their clinical application still faces challenges on multiple fronts. These concern the intrinsic biology of the markers, the design of sorting strategies, analytical technologies, and cross-tissue applicability. Progress on these fronts is gradually moving the field from fragmented insights toward a more systematic framework.

First, dynamic marker expression directly affects sorting reliability. MSC surface profiles drift markedly with culture duration, passage number, and *in vitro* microenvironment. For example, ITGA9 enables reporter-free enrichment of SOX9^+^ chondroprogenitors, but its post-expansion stability needs validation (Xiong et al., 2025); CD34 on synovial MSCs enriches osteochondral progenitors in fresh isolates, yet is rapidly lost during expansion (Noda et al., 2022). These findings suggest that marker standards from extensively cultured cells may not reflect *in situ* function. Validating markers in low-passage or freshly isolated cells, and developing culture conditions that preserve *in situ* phenotypes, can help resolve this discrepancy.

Because single markers rarely capture functional subpopulations stably, sorting strategies are shifting to multi-marker combinations. Pairs such as CD49f^+^/CD146^+^ and MSCA-1^+^/CD36^+^ define subsets with greater precision. Moreover, Chen et al. showed that BAMBIhighMFGE8high umbilical cord MSCs perform poorly in adipogenesis and immunosuppression, indicating that even positively selected populations may contain functionally inferior cells that compromise efficacy (Chen et al., 2022). Hence, incorporating negative-selection markers into multi-parameter strategies that combine positive and negative sorting can further improve product uniformity.

Implementing these strategies depends heavily on advanced tools. Single-cell multi-omics unbiasedly reveals the full landscape of MSC heterogeneity and simultaneously nominates candidate markers at transcriptomic, epigenomic, and proteomic levels. High-parameter flow cytometry then validates dozens of parameters at single-cell resolution, rapidly translating discoveries into actionable sorting schemes. Together, they form an efficient closed loop from discovery to validation, substantially shortening the cycle from laboratory screening to process development and establishing the technical feasibility required for complex multi-marker strategies.

Notably, the same functional marker may indicate divergent functional biases in MSCs from different tissues. For example, CD146 marks osteogenesis and perivascular identity in bone marrow MSCs (Harkness et al., 2016), but its role in umbilical cord MSCs is debated, with reports of adipogenic, angiogenic, or immunomodulatory activity (Wu et al., 2016; Zhang, L. et al., 2023). Similarly, CD271 identifies osteochondral progenitors in bone marrow MSCs, yet in adipose-derived MSCs its expression associates with angiogenesis, adipogenesis and immunomodulation (Hermida-Gómez et al., 2011; Calabrese et al., 2015; Smith et al., 2021; Sun et al., 2022). These observations highlight that a marker’s functional meaning is tissue-context-dependent and cannot simply be extrapolated across MSC origins. Tissue source must therefore be carefully considered when applying markers or comparing studies.

The above issues, viewed in progression, converge on a central realization that research on functional surface markers has moved beyond the discovery of individual markers into an era of multidimensional, systematic integration. Given an increasingly rich but fragmented collection of marker discoveries, a function-oriented framework that unifies these scattered pieces of evidence under a coherent perspective is urgently needed. Centered on six major types of regeneration, this article systematically delineates the mechanisms of key functional surface markers and their subpopulations. It further summarizes therapeutic advances organized by disease category, including neurological diseases, circulatory system disorders, skin and mucosal wounds, bone and joint diseases and hepatobiliary diseases, providing an evidence map for marker selection across clinical scenarios and advancing MSC therapy toward precision decisions based on functional surface markers.
